# Growth rate per year of a soft tissue recurrent ameloblastoma

**DOI:** 10.1002/ccr3.8444

**Published:** 2024-01-21

**Authors:** Roberta Rayra Martins Chaves, Wagner Henriques Castro, Ricardo Santiago Gomez

**Affiliations:** ^1^ Department of Oral Surgery and Pathology, School of Dentistry Universidade Federal de Minas Gerais Belo Horizonte Brazil; ^2^ Medical School Faculdade Ciências Médicas de Minas Gerais Belo Horizonte Brazil

**Keywords:** ameloblastoma, biological behavior, growth, odontogenic tumors

## Abstract

**Clinical Key Message:**

We present a case of recurring ameloblastoma in soft tissue, for which we have estimated the growth rate of the lesion. This information could help clinicians to establish follow‐up protocols for the early diagnosis of recurrent ameloblastomas.

**Abstract:**

In the present paper, we present a case of recurring ameloblastoma in soft tissue, for which we have estimated the growth rate of the lesion. The area of the whole resected specimen was measured using the ImageJ guide for complex object area. After dividing the area of the recurrent tumor by the number of years during the follow‐up, we found that the lesion growth rate was 5.3 cm^2^ per year. Although further studies are still necessary in the literature to assess the growth rate of ameloblastoma, the present report shows a different methodology to estimate it. This information could help clinicians to establish follow‐up protocols for the early diagnosis of recurrent ameloblastomas.

## Graphical abstract

Macroscopic view of an ameloblastoma specimen.

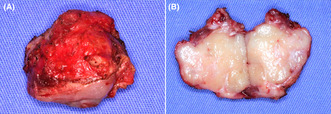

Although the growth rate of ameloblastoma is a relevant topic concerning its biological behavior, few studies in the literature have attempted to estimate it, and they were mainly based on patients' perceptions of symptoms or radiological images. In the present paper, we present a case of recurring ameloblastoma in soft tissue, for which we have estimated its growth rate in mm^2^ per year.

## 1 CASE REPORT

**FIGURE 1:**
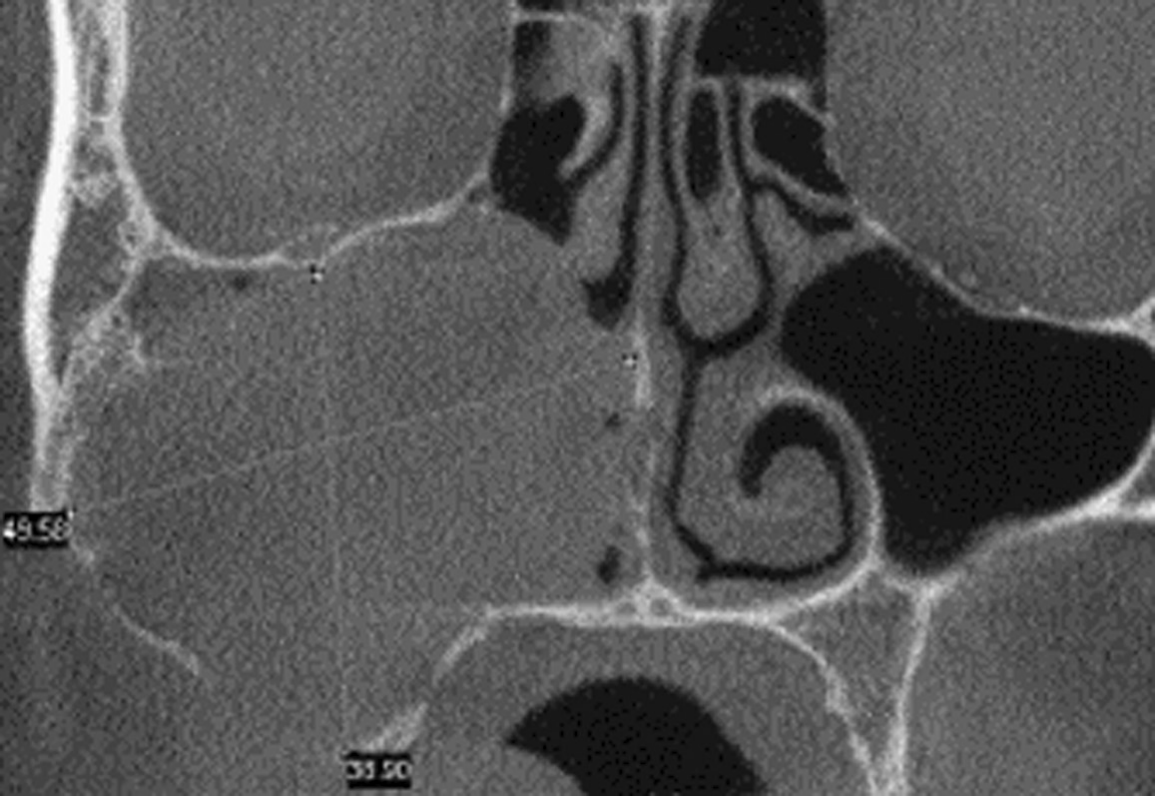
Computerized tomography showing a well‐defined, unilocular hypodense lesion in the right maxilla involving the zygomatic bone, maxillary sinus, and nasal cavity, measuring 39 × 49 56 × 47 mm.

**FIGURE 2:**
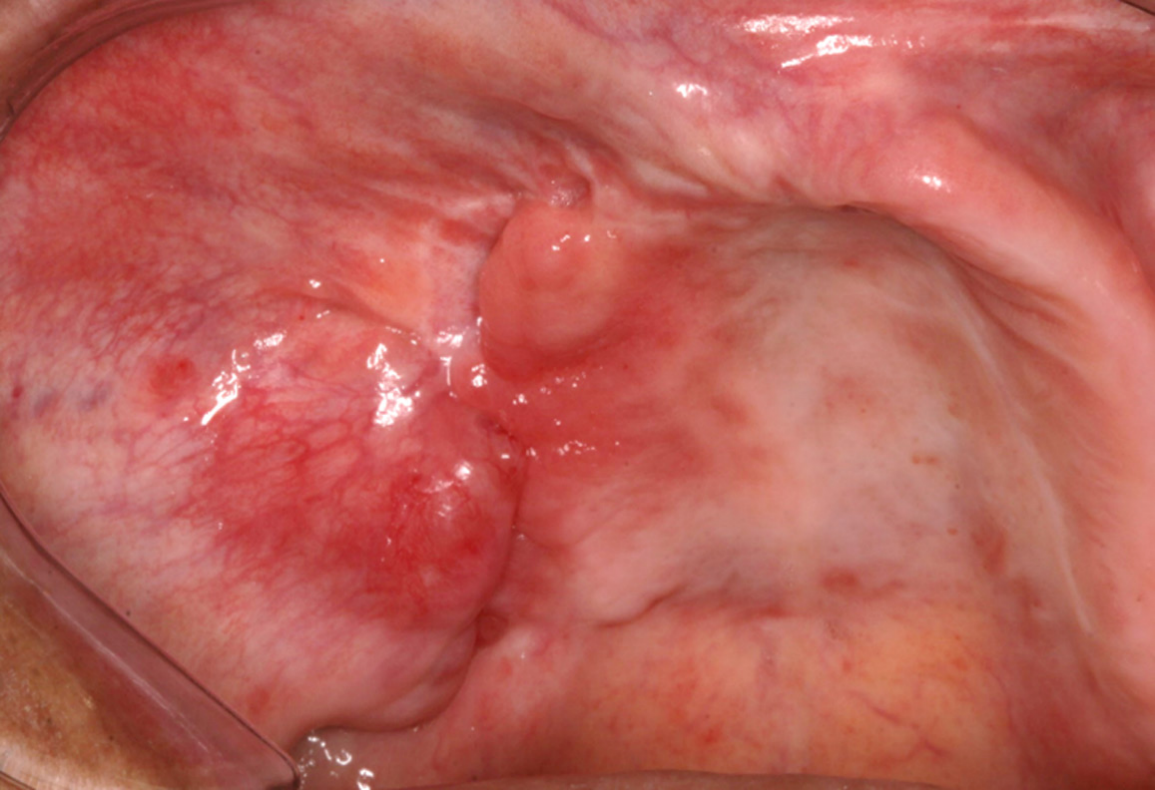
Soft tissue recurrent ameloblastoma affecting the buccal mucosa. Note the maxillary defect and scar due to the previous surgery for primary tumor removal.

**FIGURE 3:**
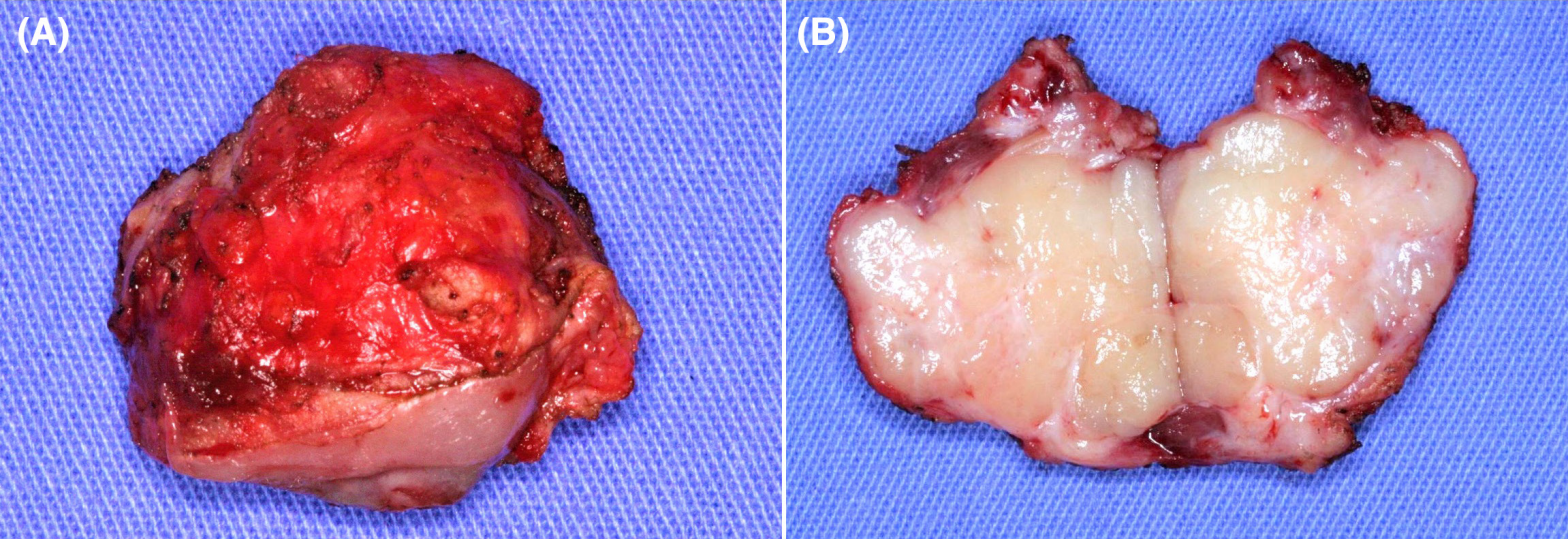
Macroscopic view of the excised specimen showing a solid and yellowish‐white macroscopic appearance.

**FIGURE 4:**
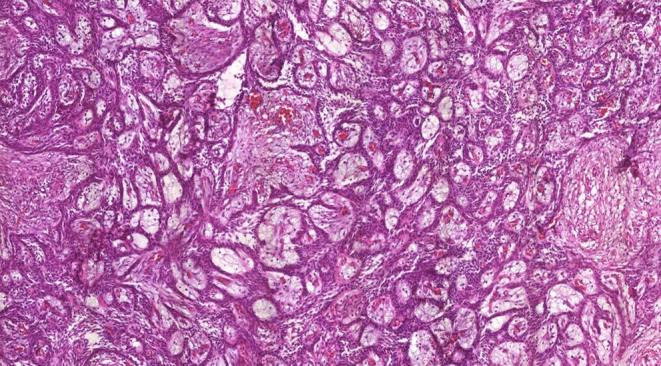
Histopathologic picture of recurrent tumor confirming the diagnosis of ameloblastoma (original magnification, H&E staining, ×20).

A 48‐year‐old male was referred to our Oral Maxillofacial Service with a chief complaint of an asymptomatic swelling in the right infraorbital region, extending to the maxillary vestibule, which was firm to palpation. Computerized tomography revealed a well‐defined, unilocular hypodense lesion in the right maxilla involving the zygomatic bone, maxillary sinus, and nasal cavity, measuring 39 × 49 56 × 47 mm (Figure 1). The incisional biopsy revealed the diagnosis of ameloblastoma, conventional subtype. Hemi‐maxillectomy was performed under general anesthesia, followed by chemical cauterization with Carnoy solution. After 6 years of follow‐up, the patient presented a tumoral lesion in the buccal mucosa without bone involvement (Figure 2). The lesion was firm to palpation, and it was asymptomatic. After tumor resection, the histopathological diagnosis was consistent with recurrent ameloblastoma (Figures 3 and 4). We used the ImageJ software to calculate the area of the surgical mass (Figure 3), which was 32 cm^2^. After 4 years of follow‐up, there was no evidence of recurrence.

## 2 DISCUSSION

Few reports in the literature attempted to evaluate the growth rate of ameloblastoma. Mariz et al.^1^ used the ImageJ software to estimate the tumor growth rate through the area measurement of matching radiographs of ameloblastomas firstly underdiagnosed. They found that the tumor grows an average of 40.4% per year (14.9–88.7%). Although intriguing, the usual distortion observed in panoramic radiographs can alter the tumor's actual size, limiting this study's findings.

Odukoya & Effiom^2^ attempted to estimate the growth rate of ameloblastoma by correlating the tumor volume with the duration of the symptoms. They showed a growth rate of 0.81 cm^3^/month (9.72 cm^3^/year) for conventional and 0.17 cm^3^/month (2.04 cm^3^/year) for the unicystic subtype. Effiom & Odukoya^3^ used the same method to find that desmoplastic ameloblastomas grow an average of 0.7 cm/month. Despite the valuable information, the duration of symptoms reported by patients on clinical evaluation was used to calculate the time to tumor progression, which may have been inaccurate. A systematic review also found a growth rate of 87.8% per year for ameloblastoma,^4^ but again the period used in all studies was based on the patient's perception of symptoms.

Although recurrence in ameloblastoma is not an unusual feature, authors usually do not report their dimension together with the period of follow‐up in their studies. The fact that ameloblastoma is an intraosseous disease makes it difficult to retrieve information regarding its initial development. As nearly all recurrences occur inside the bone, it is also challenging to estimate their dimension radiologically because of the previous destruction of bone caused by the primary tumor and the surgical procedures for its resection.

In the present report, the recurrence occurred inside the soft tissues of the buccal mucosa, allowing the careful dissection of the tumor, which is impossible in intraosseous lesions. Then, we estimated the growth rate of the lesion by measuring the area of the whole resected specimen following the ImageJ guide for complex object area measurement^5^ and dividing it by the follow‐up period in years from the removal of the primary tumor to the recurrence diagnosis. Using this method, we found that the lesion growth rate was 5.3 cm^2^ per year. The methodological difference in evaluating a soft tissue recurrence may explain the discrepancy between our findings and that of previous studies. Although further studies are still necessary in the literature to assess the growth rate of ameloblastoma, the present report shows a different methodology to estimate it.

## AUTHOR CONTRIBUTIONS

**Roberta Rayra Martins Chaves:** Conceptualization; data curation; formal analysis; investigation; methodology; writing – original draft; writing – review and editing. **Wagner Henriques Castro:** Conceptualization; formal analysis; investigation; methodology; writing – review and editing. **Ricardo Santiago Gomez:** Conceptualization; formal analysis; investigation; methodology; project administration; writing – original draft; writing – review and editing.

## CONSENT

Written informed consent was obtained from the patient to publish this report in accordance with the journal's patient consent policy.

## Data Availability

Data available on request from the authors.
